# Comparative decline in funding of European Commission malaria vaccine projects: what next for the European scientists working in this field?

**DOI:** 10.1186/1475-2875-10-255

**Published:** 2011-09-01

**Authors:** Regitze L Thøgersen, Anthony A Holder, Adrian VS Hill, David E Arnot, Egeruan B Imoukhuede, Odile Leroy

**Affiliations:** 1 European Vaccine Initiative, UniversitätsKlinikum Heidelberg, Im Neuenheimer Feld 326, 69120 Heidelberg, Germany; 2 MRC National Institute for Medical Research, Division of Parasitology, The Ridgeway, Mill Hill, London NW7 1AA, UK; 3 University of Oxford, Jenner Institute, Old Road Campus Research Building, Roosevelt Drive, Oxford, OX3 7DQ, UK; 4 Copenhagen University, Centre for Medical Parasitology, Bartholinsgade 2, 1356 Copenhagen K, Denmark

## Abstract

Since 2000, under the Fifth and subsequent Framework Programmes, the European Commission has funded research to spur the development of a malaria vaccine. This funding has contributed to the promotion of an integrated infrastructure consisting of European basic, applied and clinical scientists in academia and small and medium enterprises, together with partners in Africa. Research has added basic understanding of what is required of a malaria vaccine, allowing selected candidates to be prioritized and some to be moved forward into clinical trials. To end the health burden of malaria, and its economic and social impact on development, the international community has now essentially committed itself to the eventual eradication of malaria. Given the current tentative advances towards elimination or eradication of malaria in many endemic areas, malaria vaccines constitute an additional and almost certainly essential component of any strategic plan to interrupt transmission of malaria. However, funding for malaria vaccines has been substantially reduced in the Seventh Framework Programme compared with earlier Framework Programmes, and without further support the gains made by earlier European investment will be lost.

## Background

Approximately one million people die each year from malaria, mostly African children under the age of five and pregnant women [1,2]. Malaria is, therefore, one of the major global killer diseases and one of the world's biggest public health problems [1,3]. As malaria is mainly prevalent in low-income countries, it places a disproportionately heavy economic burden on endemic countries, contributing to poverty and limiting economic development [1,4].

Malaria is a complex disease, which is transmitted to humans through the bite of the female *Anopheles *mosquito inoculating protozoan parasites of the genus *Plasmodium*. There are five types of human malaria, *Plasmodium falciparum, Plasmodium vivax, Plasmodium malariae, Plasmodium ovale*, and *Plasmodium knowlesi*, with *P. falciparum *and *P. vivax *being the most common and *P. falciparum *being the most deadly [1,2].

A protective malaria vaccine that prevents or reduces clinical malaria and associated mortality, as well as reducing transmission, will have a major impact on global human health and socioeconomic development. There is considerable optimism that a malaria vaccine can be developed, as immunity developed following natural infection eventually prevents mortality and protects against clinical disease [3].

A malaria vaccine developed by GlaxoSmithKline (GSK) is being tested in various African countries in Phase III clinical trials, following earlier relatively promising results indicating that perhaps 40-50% of immunized children were protected against natural infection for a significant period [5]. However, it seems unlikely that GSK's RTS, S pre-erythrocytic stage vaccine will generate higher levels of protection in Phase III clinical trials than those achieved in Phase II clinical trials and it is doubtful whether the RTS, S vaccine will have any impact on reducing malaria transmission. To achieve elimination and eventual eradication, it is clearly necessary to intensify the research and development effort to obtain more protective, longer lasting malaria vaccines, including vaccines which also protect against other species of malaria, particularly the very widely transmitted *P. vivax*.

### History of European Commission funded malaria vaccine projects

Under the Fifth Framework Programme (FP) plan for Research and Technology Development (1998-2002), the European Commission (EC) funded a broad range of research activities related to the three major poverty-related diseases (PRDs), HIV/AIDS, malaria and tuberculosis. A total of 24 malaria projects were supported in this period, with a total budget of approximately € 29 million, of which around € 17 million was dedicated to malaria vaccine projects [1,6]. Increased funding for PRD research was provided in FP6 (2002-2006) and a portfolio of 17 malaria projects was established, with an overall budget of about € 64 million, of which almost € 20 million was dedicated to malaria vaccine projects [1].

In the current FP7 plan, (2007-2013), efforts were made to capitalize on the advances achieved under FP6 by improved integration of basic malaria research with more robust research and management structures to translate research results into therapeutic drugs or vaccine candidates. By the end of 2010, halfway through the FP7, a total of nearly € 80 million was earmarked for malaria research. However, only around € 10 million was earmarked for malaria vaccine research [1]. This probably reflected optimism that the GSK RTS, S vaccine would translate well into the developing global anti-malaria campaign and fuelled suggestions that improved vaccines were not a priority.

The past and present EC funded malaria vaccine projects under FP5, FP6 and FP7 are listed in Table 1, 2 and 3 respectively [1].

**Table 1:** Malaria vaccine projects funded by the EC FP5

Project	Project	Coordinator	Partner	EC Contribution	Duration	Start
Acronym	Type	Name/Institution	Numbers	Euros	Months	Date
EUROMALVAC1	RS	D. Arnot, University of Edinburgh, UK	10	3 500 000	36	01-02-2000
ATTMAL	RS	A. Waters, Leiden University, NL	5	1 492 640	36	01-03-2000
MALTRANS	RS	R. Sauerwein, Radboud University Nijmegen Medical Centre, NL	11	2 499 962	36	01-03-2000
AMVTNETHIC	AM	S. Jepsen, Statens Serum Institut, DK	1	220 000	36	01-04-2000
PAMVAC	RS	M. Klinkert, Bernhard-Nocht-Institute for Tropical Medicine, DE	6	1 503 210	36	01-09-2001
NEMLAR	AM	S. Jepsen, Statens Serum Institut, DK	1	70 000	36	01-12-2001
EMLI	DM	P. Druilhe, Institut Pateur, FR	7	1 462 733	36	01-01-2002
EMVI	AM	S. Jepsen, Statens Serum Institut, DK	2	700 000	36	01-08-2002
EUROMALVAC2	RS	D. Arnot, University of Edinburgh, UK	12	3 700 044	36	01-09-2002
VIRIMAL	RS	P. Preiser, Medical Research Council, UK	5	1 196 180	36	01-09-2002
AMVTN/AMANET	CA	S. Jepsen, Statens Serum Institut, DK	16	1 000 000	36	01-11-2002

***Total EC contribution under FP5***				***17 344 769***		

AM: Accompanying Measures, CA: Coordination Action, DM: Demonstration, RS: Research

**Table 2:** Malaria vaccine projects funded by the EC FP6

Project	Project	Coordinator	Partner	EC Contribution	Duration	Start
Acronym	Type	Name/Institution	Numbers	Euros	Months	Date
MALINV	STREP	L. Rénia, Département d'Immunologie, Institut Cochin, FR	5	587 000	24	01-06-2005
SME Malaria	STREP	R. Glück, Etna Biotech, IT	5	1 700 000	36	01-03-2006
EMVDA	IP	O. Leroy, European Vaccine Initiative, DE	15	13 500 000	63	01-12-2006
EURHAVAC	SSA	O. Leroy, European Malaria Vaccine Initiative, DK	1	260 000	24	01-12-2006
CILMALVAC	STREP	M. Hartmann, Cilian AG, DE	3	1 271 664	36	01-01-2007
PRIBOMAL	STREP	J. Goudsmit, Crucell Holland, NL	7	2 345 358	48	01-02-2007

***Total EC contribution under FP6***				***19 664 022***		

IP: Integrated Project, SSA: Specific Support Actions, STREP: Specific Targeted Research Project

**Table 3:** Malaria vaccine projects funded by the EC FP7

Project	Project	Coordinator	Partner	EC Contribution	Duration	Start
Acronym	Type	Name/Institution	Numbers	Euros	Months	Date
PreMalStruct	S/M-SFRP	B. Gamain, Institut Pasteur, FR	5	2 300 000	36	01-02-2008
STOPPAM	S/M-SFRP	M. Laurent, Institut de Recherche Pour le Development, FR	6	3 000 000	36	01-02-2008
INYVAX	CA	O. Leroy, European Vaccine Initiative, DE	7	932 335	36	01-02-2009
OPTIMALVAC	CA	O. Leroy, European Vaccine Initiative, DE	12	1 000 000	36	01-04-2009
REDMAL	FRP	R. Sauerwein, Radboud University Nijmegen Medical Centre, NL	6	2 999 998	48	01-03-2010

***Total EC contribution under FP7***				***10 232 333***		

CA: Coordination Action, S/M-SFRP: Small or medium-scale focused research project

### Key successes, their impact and future perspectives

Vaccines are one of the most effective ways to protect people against infectious diseases, and one of the most cost-effective measures of public health. A malaria vaccine is unlikely to be developed if not supported by public sector and charitable funding and international public organizations, such as the EC [7].

Useful and significant advances have already been achieved by several malaria vaccine projects funded by the EC. A number of its malaria vaccine development projects are in the clinical testing process and will report in the next period. Current EC strategies clearly complement those of other international agencies in this area and have the potential to make a major impact on malaria vaccine development projects. It is, therefore, critical that the EC continues to build on the major advances in understanding immunity to malaria that have been achieved and on several successes in raising immunogenicity of candidate vaccines. Rather than abandon this field, mechanisms for continuing to develop more effective malaria vaccines by building on the current RTS, S successes should be established [7].

## Conclusions

To maintain and consolidate the European vaccine development infrastructure and exploit the considerable depth of European expertise in this area, continued funding for malaria vaccine research is essential. Doing otherwise will ensure that European vaccine R&D teams, in both industry and academia, will be forced to disband and Europe risks becoming irrelevant in the global push to eliminate malaria, just as this campaign gathers momentum.

## Competing interests

The authors declare that they have no competing interests.

## Authors' contributions

RLT, project manager of the EMVDA project, has coordinated and contributed to the writing of the manuscript. OL, coordinator of the EMVDA project and EBI, clinical trial director, have contributed to the writing of the manuscript. AAH, AVSH and DEA have participated in a number of EC-funded malaria vaccine development projects and have contributed to the writing of the manuscript. All authors read and approved the final manuscript.
